# MHC2SKpan: a novel kernel based approach for pan-specific MHC class II peptide binding prediction

**DOI:** 10.1186/1471-2164-14-S5-S11

**Published:** 2013-10-16

**Authors:** Linyuan Guo, Cheng Luo, Shanfeng Zhu

**Affiliations:** 1 School of Computer Science and Shanghai Key Lab of Intelligent Information Processing, Fudan University, Shanghai 200433, China

## Abstract

**Background:**

Computational methods for the prediction of Major Histocompatibility Complex (MHC) class II binding peptides play an important role in facilitating the understanding of immune recognition and the process of epitope discovery. To develop an effective computational method, we need to consider two important characteristics of the problem: (1) the length of binding peptides is highly flexible; and (2) MHC molecules are extremely polymorphic and for the vast majority of them there are no sufficient training data.

**Methods:**

We develop a novel string kernel MHC2SK (MHC-II String Kernel) method to measure the similarities among peptides with variable lengths. By considering the distinct features of MHC-II peptide binding prediction problem, MHC2SK differs significantly from the recently developed kernel based method, GS (Generic String) kernel, in the way of computing similarities. Furthermore, we extend MHC2SK to MHC2SKpan for pan-specific MHC-II peptide binding prediction by leveraging the binding data of various MHC molecules.

**Results:**

MHC2SK outperformed GS in allele specific prediction using a benchmark dataset, which demonstrates the effectiveness of MHC2SK. Furthermore, we evaluated the performance of MHC2SKpan using various benckmark data sets from several different perspectives: Leave-one-allele-out (LOO), 5-fold cross validation as well as independent data testing. MHC2SKpan has achieved comparable performance with NetMHCIIpan-2.0 and outperformed NetMHCIIpan-1.0, TEPITOPEpan and MultiRTA, being statistically significant. MHC2SKpan can be freely accessed at http://datamining-iip.fudan.edu.cn/service/MHC2SKpan/index.html.

## Background

Binding of antigenic peptides to major histocompatibility complex (MHC) class molecules is a core step in adaptive (specific) immune response. There are two major categories of MHC molecules: class I MHC (MHC-I) molecules and class II MHC (MHC-II) molecules. In contrast to MHC-I that mainly recognize peptides from intracellular antigens, MHC-II molecules are mainly responsible for binding peptides from extracellular antigens. These binding peptides are then presented on cell surfaces to the receptors of T helper (Th) cells, by which the adaptive immune system recognizes the antigen and starts specific responses, such as activating B cells to excrete antibodies neutralizing the pathogen [1]. Therefore, the accurate prediction of MHC binding peptides is important for understanding the mechanism of immune recognition and facilitating the process of epitope based vaccine design [2]. With the advantage of low financial cost and rapid deployment, computational methods have become increasingly important. They have already been used to choose very few promising candidate eptiopes that are further verified by biochemical experiments [3].

Although many computational methods have been developed to predict MHC class II binding peptides in the last few years [4-15], recent experimental results on benchmark datasets show that the performance of these methods needs to be improved [16-18]. Two distinct characteristics make the MHC-II peptide binding prediction problem very difficult. Firstly, the binding groove of MHC class II molecules is open in two directions. This results in a large length variation of of binding peptides (usually 11-20 amino acids) [19]. Several computational methods, such as TEPITOPE [9], SMM-align [4] and NN-align [5], try to locate the binding core of a peptide in the modeling process, which is a nonamer sitting in the binding groove of MHC molecules. However, the identified core may not be accurate and other important sequence information would be lost. Secondly, MHC are extremely polymorphic with a few thousand allele variants. By October 2012, IMGT/HLA has accumulated more than 1800 HLA (human leukocyte antigen, the name of MHC in Humans) class II allelic variants [20]. Many earlier computational methods, such as SMM-align and NN-align, are allele-specific ones that use the binding data of target MHC molecule to train a model to predict its binding specificity. However, vast majority of MHC-II molecules do not have sufficient binding data to train a reliable prediction model. In fact, there are less than 35 HLA class II molecules that have several hundred peptides with binding affinities in IEDB [21]. For addressing this problem, pan-specific approaches have been recently proposed to make predictions for any alleles with the known protein sequence [18]. The basic idea of pan-specific methods is to identify the relationship among MHC alleles so that the binding preferences of target MHC molecules can be captured.

MULTIPRED is the first pan-specific predictor for HLA-I [22]. It trains a supertype-specific model by incorporating the binding data in the same supertype, where a set of MHC molecules have similar peptide binding preferences [23]. Our pervious work has shown that incorporating binding data of MHC-I molecules in the same supertype can alleviate the scarcity of binding data and improve the prediction accuracy [24]. Moreover, in the last few years, several pan-specific methods have been developed for predicting the binding specificity of MHC-II molecules based on different principles [9-15], such as position specific scoring matrices (PSSMs), artificial neural network (ANN) and kernel based method. TEPITOPE [9] and TEPITOPEpan [15] are two PSSMs based methods. TEPTIOPE is a pioneering MHC-II pan-specific predictor, with the limitation of covering only 51 out of more than 1000 HLA-DR alleles. To overcome this limitation, we have developed TEPITOPEpan that covers all possible HLA-DR alleles. Its main idea is to extrapolates the preferences of 51 HLA-DR molecules covered by TEPITOPE to all uncharacterized. Not only NetMHCIIpan-1.0 [10] but also NetMHCIIpan-2.0 [11] are ANN based methods. Both versions utilize an ensemble of artificial neural network (ANN) with different network structures and initialization parameters, while the main difference is the way of determining the binding core. MultiRTA [14] is based on a regularized thermodynamic model and it considers all possible binding core configurations. MHCIIMulti [12] is a kernel based method that makes use of multi-instance technique for measuring the similarity between peptides. According to several recent bench-mark studies, overall NetMHCIIpan-2.0 performed the best, whereas TEPITOPE and TEPITOPEpan were good at identifying binding core, and achieved good accuracy in recognizing T-cell epitopes as well as HLA-ligands [15,18].

Compared with feature vector based methods, kernel-based methods can deal with the flexibility of peptide lengths more naturally. With carefully designed kernels, these methods can perform very well without undertaking the complicated tasks of feature extraction and selection [25]. Most recently, Giguère et al. has developed a general string (GS) kernel for leaning a peptide-protein binding affinity [26], and GS kernel has achieved the good prediction accuracy in several applications, such as peptide-protein binding prediction on the data from the PepX database, MHC-II binding prediction and quantitative structure affinity prediction. The similarity between two peptides defined by GS is actually a sum of similarity scores by substring comparisons. Because GS was designed for a general problem of peptide-protein binding prediction, it did not take into consideration some distinct features of MHC-II binding peptides. Firstly, GS considers very short substrings of even one or two amino acids in computing similarity. Moreover, the consideration of long substrings for computing similarity in GS depends on its parameter. However, a short substring pattern is less significant and may bring noise, while the long substring pattern should be favored. Secondly, GS penalizes the similarity of two substrings if their starting positions in two peptides are different. However, this kind of penalization is unreasonable for MHC-II binding peptides. For example, it is common for the binding cores of two peptides starting at different positions. The similarity between these two binding cores by GS would be very low due to penalization even if they are identical. To overcome these drawbacks of GS, we propose a new string kernel for MHC-II, MHC2SK, which emphasizes the long substring of peptides and considers the variation of peptide lengths.

MHC2SK outperformed GS in the allele-specific prediction task on a benchmark dataset, which demonstrates the effectiveness of MHC2SK. Furthermore, we extended MHC2SK to MHC2SKpan for pan-specific MHC-II peptide binding prediction by leveraging the binding data of various MHC molecules. We evaluated the performance of MHC2SKpan on three benchmark datasets from several aspects: Leave-one-allele-out (LOO), 5-fold cross validation as well as independent data testing. MHC2SKpan achieved comparable performance with NetMHCIIpan-2.0 and outperformed TEPITOPEpan, NetMHCIIpan-1.0 and MultiRTA, being statistically significant.

## Materials and methods

### Data

We used 4 benchmark data sets: NielsenSet1, NielsenSet2, NielsenSet3 and EpanSet4 to evaluate the performance of different MHC-II peptide binding prediction methods. Specifically, NielsenSet1 was used for comparing the performance of MHC2SK with a kernel based allele-specific method, GS. The remaining three were used for comparing the performance of MHC2SKpan with other four well-known pan-specific predictors, such as NetMHCIIpan-2.0, NetMHCIIpan-1.0, TEPITOPEpan and MultiRTA.

NielsenSet1 consists of 4603 peptides covering 14 HLA-DR molecules. It was originally used for developing the SMM-align method [4]. NielsenSet2 was obtained from [10], and it is composed of 14607 peptides associated with 14 HLA-DR molecules. NielsenSet3 was taken from [11], and it consists of 33931 peptides covering 24 HLA-DR molecules. EpanSet4 was from [15] and was composed of 2412 peptides covering 14 HLA-DR molecules. These 14 molecules are neither in NielsenSet1, nor in NielsenSet2, with only two of them appearing in NielsenSet3. This is why the dataset was originally used for evaluating the performance of different pan-specific methods on novel MHC molecules [15].

### Method

In this section, we briefly describe several string kernels related to our work. After presenting the notations, we first introduce Spectrum RBF string kernel (SRBF), which is closely related to GS and MHC2SK. After that, we describe GS and our newly developed MHC2SK kernel. Finally, we extend MHC2SK to MHC2SKpan for pan-specific MHC-II binding prediction.

#### Notation

Let Σ be a set of all the alphabets of amino acids, and for each amino acid *a *∈ Σ we define an encoding function $\varphi :\Sigma \to {\mathbb{R}}^{d}$. *φ*(*a*) = (*φ*_1_(*a*), *φ*_2_(*a*), ..., *φ_d_*(*a*)) is a vector where *φ_i_*(*a*) represents one of the *d *properties of the amino acid *a*. In the experiments we utilize the widely used Blosum62 [27] to define the encoding function *φ*. In the following subsections we denote *s *and *s*' as two amino acid chains with length |*s*| and |*s*'| respectively. Similarly, we denote *y *and *y*' as two peptides, *yi*→*i*+*l*-1 is a substring of *y *of length *l *with the starting position *i *and end position *i *+ *l *- 1, *y*'_*j*→*j*+*l*-1 _is a substring of *y' *of length *l *with the starting position *j *and end position *j *+ *l *- 1, and *x *and *x' *as two MHC molecules (or its pseudosequence representation).

#### Spectrum RBF string kernel (SRBF)

The spectrum RBF string kernel was proposed by Toussaint et al. [28] for MHC-I peptide binding prediction. As spectrum RBF string kernel is directly related to GS and MHC2SK, we review it briefly here. For *s *and *s' *with an equal length under a certain encoding scheme, such as Blosum62, we can compute their similarity using RBF kernel

(1) $$K_{l,\sigma_{c}}^{\varphi }\left(s,s^{\prime }\right)=\text{exp}\left(-\frac{\sum_{i=1}^{l}||\varphi \left(s_{i}\right)-\varphi \left({s^{\prime }}_{i}\right)|{|}^{2}}{2\sigma_{c}^{2}}\right)$$

where |*s*|=|*s*'|=*l *and *s_i _*denote the *i*-th amino acid in sequence *s*. Similar to spectrum kernel [29], the similarity between two peptides *y *and *y' *with different lengths can be computed by considering the substrings of length *l*. According to [28], SRBF can be computed as follows

(2) $$K_{SRBF}\left(y,y^{\prime },l,\sigma_{c}\right)≜\overset{|y|-l+1}{\underset{i=1}{\sum }}\overset{|y^{\prime }|-l+1}{\underset{j=1}{\sum }}K_{l,\sigma_{c}}^{\varphi }\left(y_{i\to i+l-1},y_{j\to j+l-1}^{\prime }\right)=\overset{|y|-l+1}{\underset{i=1}{\sum }}\overset{|y^{\prime }|-l+1}{\underset{j=1}{\sum }}\text{exp}\left(-\frac{\sum_{k=0}^{l-1}||\varphi \left(y_{i+k}\right)-\varphi \left({y^{\prime }}_{j+k}\right)|{|}^{2}}{2\sigma_{c}^{2}}\right)$$

where *y*_*i*+*k *_denote the (*i *+ *k*)-th amino acid in the sequence *y*. It's worth noticing that, for computing the similarity between *y *and *y'*, *K_S RBF _*only compares their substrings with a fixed length (*l*), which may ignore some important information about the commonality of *y *and *y'*.

#### Generic String kernel (GS)

GS was proposed by Giguère et al. as a general kernel for learning peptide-protein binding [26]. It can be formulated as follows:

(3) $$\begin{array}{llll} K_{GS}\left(y,y^{\prime },L,\sigma_{p},\sigma_{c}\right) & ≜\overset{L}{\underset{l=1}{\sum }}\overset{|y|-l+1}{\underset{i=1}{\sum }}\overset{|y^{\prime }|-l+1}{\underset{j=1}{\sum }}\text{exp}\left(\frac{-{\left(i-j\right)}^{2}}{2\sigma_{p}^{2}}\right)K_{l,\sigma_{c}}^{\varphi }\left(y_{i\to i+l-1},{y^{\prime }}_{j\to j+l-1}\right)\; & & \;\\ & =\overset{L}{\underset{l=1}{\sum }}\overset{|y|-l+1}{\underset{i=1}{\sum }}\overset{|y^{\prime }|-l+1}{\underset{j=1}{\sum }}\text{exp}\left(\frac{-{\left(i-j\right)}^{2}}{2\sigma_{p}^{2}}\right)\text{exp}\left(-\frac{\sum_{k=0}^{l-1}||\varphi \left(y_{i+k}\right)-\varphi \left({y^{\prime }}_{j+k}\right)|{|}^{2}}{2\sigma_{c}^{2}}\right)\; & & \;\\ & \; & \end{array}$$

where *L *≥ 1 is the maximum length of substrings under comparison, and *σ_p _*is the parameter for penalizing the similarity of *y *and *y*'_*j*→ *j*+*l*-1 _that start from different positions of i and j, respectively. From this, we can see that GS is a weighted combination of many SRBFs that take into account substrings with different lengths. However, considering the distinct features of MHC-II binding prediction, the penalization is unreasonable, and an additional parameter *σ_p _*also increases the training time significantly. In addition, GS considers SRBFs of very short substrings, only one amino acid (*l *= 1 in equation (3)). This kind of short patterns are less significant, and may bring noise into the similarity computation.

#### MHC-II String Kernel (MHC2SK)

Considering the distinct features of MHC-II binding prediction, we design a novel kernel, MHC2SK, as follows

(4) $$\begin{array}{llll} K_{M\;HC2S\;K}\left(y,y^{\prime },L,\sigma_{c}\right) & ≜\overset{\text{min}\left(\left|y|,|y^{\prime }\right|\right)}{\underset{l=L^{\prime }}{\sum }}\overset{|y|-l+1}{\underset{i=1}{\sum }}\overset{|y^{\prime }|-l+1}{\underset{j=1}{\sum }}K_{l,\sigma_{c}}^{\varphi }\left(y_{i\to i+l-1},{y^{\prime }}_{j\to j+l-1}\right)\; & & \;\\ & =\overset{\text{min}\left(\left|y|,|y^{\prime }\right|\right)}{\underset{l=L^{\prime }}{\sum }}\overset{|y|-l+1}{\underset{i=1}{\sum }}\overset{|y^{\prime }|-l+1}{\underset{j=1}{\sum }}\text{exp}\left(-\frac{\sum_{k=0}^{l-1}||\varphi \left(y_{i+k}\right)-\varphi \left({y^{\prime }}_{j+k}\right)|{|}^{2}}{2\sigma_{c}^{2}}\right)\; & & \;\\ & \; & \end{array}$$

There are two main differences between MHC2SK and GS. Firstly, MHC2SK removes the penalized term $\text{exp}\left(\frac{-{\left(i-j\right)}^{2}}{2\sigma_{p}^{2}}\right)$ in the similarity computation. Omitting the parameter *σ_p _*also reduces the training cost significantly. Secondly, MHC2SK emphasizes more on longer substring patterns for computing similarity. *L*' is the parameter for the minimum length of substring patterns considered in MHC2SK, while the maximum length is the largest possible length (*min*(|*y*|, |*y*'|)). In contrast, the minimum length of substring patterns in GS is 1, and the maximum length is determined by *L*. We can see that MHC2SK is a combination of SRBFs considering different lengths, thus MHC2SK is also positive semi-definite.

#### MHC-II String Kernel for pan-specific prediction (MHC2SKpan)

For the purpose of training a pan-specific model for any alleles with the known protein sequence, similar to the strategy proposed by KISS [30], we define the allele-peptide (*x*, *y*) pairwise kernel by obtaining the product between an allele kernel and a peptide kernel.

(5) $$K\left(\left(x,y\right),\left(x^{\prime },y^{\prime }\right)\right)\;≜\;K_{allele}\left(x,x^{\prime }\right)\;\cdot K_{peptide}\left(y,y^{\prime }\right)$$

For the peptide kernel, we can use MHC2SK kernel. For the HLA allele representation, we apply the pseudo sequence proposed by Nielsen et al [10]. The pseudo sequence is composed of 21 polymorphic amino acid positions in potential contact with the binding peptide. Since all the allele pseudo sequences are of equal length, we use the RBF kernel (equation 1) as the allele kernel. Then we can extend MHC2SK to MHC2SKpan for pan-specific prediction as follows:

(6) $$K_{MHC\text{2}S\;K\;pan}\left(\left(x,y\right),\left(x^{\prime },y^{\prime }\right)\right)\;≜\;K_{allele}\left(x,x^{\prime }\right)\cdot K_{peptide}\left(y,y^{\prime }\right)=K_{|x|,\sigma_{a}}^{\varphi }\left(x,x^{\prime }\right)\cdot K_{MHC\text{2}S\;K}\left(y,y^{\prime },L^{\prime },\;\sigma_{c}\right)$$

where |*x*| = |*x*'| is the length of HLA pseudo sequence (21 in our case).

## Results and discussion

### Experimental procedure and evaluation metrics

The prediction model was learned by the support vector regression (SVR) algorithm. We made use of libsvm tool [31] and its SVR implementation with customized kernels, which were computed by the methods mentioned in the last section. The libsvm tool can be downloaded at http://www.csie.ntu.edu.tw/~cjlin/libsvm/. Two standard metrics, the area under ROC curve (AUC) and Pearson correlation coefficient (PCC), were used to evaluate the performance of different prediction methods. In addition, for comparing performance differences of two predictors, we use one-tailed per-allele binomial test to measure its statistical significance.

For the datasets of NielsenSet1, NielsenSet2 and NielsenSet3, according to the studies presenting these data [4,10,11], the peptide with the binding affinity of less than 500nM was deemed as a binder. For EpanSet4, binding affinity is not available, and we used the binary labels in the dataset directly. Similar to several previous studies [4,10], for computing PCC, the binding value was obtained by 1 - *log*(*IC*50)/*log*(50, 000), where IC50 is binding affinity measured in nM. We first compared the performance of GS and MHC2SK using NielsenSet1 by 5-fold cross validation. As SRBF is closely related to GS and MHC2SK, we also implemented SRBF as a baseline. We then compared the performance of MHC2SKpan with several well-known pan-specific methods, using Leave-One-Allele-Out (LOO) on NielsenSet2 and 5-fold cross validation on NielsenSet3. Finally we examined the performance of MHC2SKpan and other pan-specific methods on an independent test set, EpanSet4. These experiments have different focuses. The main purpose of LOO is to examine the generalization ability of pan-specific methods on novel alleles. For the 5-fold cross validation, the main purpose is to examine the performance of pan-specific methods using binding data of both target and other alleles. For the independent test, the main purpose is to examine the performance of pan-specific methods on the test data from different sources. For all the experiments, we used the grid search to learn the parameters in the three kernels. For GS kernel, we used the following ranges: *σ_p _*∈ (0, 15], *σ_c _*∈ (0, 5] and *L *∈ [1,20]. For MHC2SK kernel, we used the following ranges: *σ_c _*∈ (0, 5] and *L*' ∈ [1,9]. Compared with MHC2SK, MHC2SKpan had an additional parameter *σ_a_*, which was searched in (0, 15]. For SRBF kernel, we used the following ranges: *σ_c _*∈ (0, 5] and *l *∈ [1,9].

### Evaluation by NielsenSet1

Table 1 shows the performance comparison of MHC2SK, GS and SRBF on NielsenSet1 using 5-fold cross validation. We obtain the 5 fold partition of the data from the original study [4]. Same as [4], in each round, 4 folds are used for training the model and tuning the parameters according to AUC. The best parameters on training data are used to build the model and make the prediction on test data. As illustrated in Table 1, MHC2SK achieved the best performance in both AUC and PCC. For example, MHC2SK achieved the highest average PCC of 0.450, which is followed by SRBF (0.419) and GS (0.411). Specifically, MHC2SK outperformed GS in 12 and SRBF in 11 out of all 14 alleles. Both of them are statistically significant (binomial test, *p*-value < 0.05). In addition, MHC2SK obtained the highest average AUC (0.747), which is followed by GS (0.727) and SRBF (0.718). Specifically, MHC2SK outperformed SRBF in 11 out of all 14 alleles, being statistically significant (binomial test, *p*-value < 0.05), and GS in 9 out of all 14 alleles. From the experimental results, we can clearly see that MHC2SK performed best among all three kernel based methods.

**Table 1:** Five-fold cross validation performance of MHC2SK method compared to GS and SRBF methods on NielsenSet1. For each allele, we display the largest value in boldface.

		AUC			PCC		
**allele**	**count**	**SRBF**	**GS**	**MHC2SK**	**SRBF**	**GS**	**MHC2SK**

DRB1*01:01	1203	0.766	0.791	**0.804**	0.504	0.519	**0.559**
DRB1*03:01	474	**0.755**	0.712	0.735	**0.475**	0.423	0.473
DRB1*04:01	457	0.728	**0.761**	0.754	0.428	**0.490**	0.481
DRB1*04:04	168	**0.769**	0.653	0.757	**0.415**	0.254	0.411
DRB1*04:05	171	0.683	0.648	**0.709**	0.409	0.273	**0.430**
DRB1*07:01	310	0.773	0.745	**0.775**	0.502	0.464	**0.513**
DRB1*08:02	174	0.766	**0.783**	0.782	0.452	0.461	**0.485**
DRB1*09:01	117	0.623	0.656	**0.661**	0.290	0.269	**0.339**
DRB1*11:01	359	0.724	0.737	**0.774**	0.427	0.463	**0.518**
DRB1*13:02	179	0.846	0.817	**0.848**	**0.663**	0.617	0.662
DRB1*15:01	365	0.798	0.786	**0.801**	0.582	0.566	**0.586**
DRB3*01:01	102	0.428	**0.660**	0.650	-0.082	**0.147**	0.015
DRB4*01:01	181	0.716	**0.743**	0.738	0.437	0.447	**0.465**
DRB5*01:01	343	0.681	**0.688**	0.675	0.363	0.363	**0.365**

average	4603	0.718	0.727	**0.747**	0.419	0.411	**0.450**

### Evaluation by NielsenSet2

Table 2 presents the result of MHC2SKpan and four other well-known predictors, MultiRTA, TEPITOPEpan, NetMHCIIpan-2.0 and NetMHCIIpan-1.0 using NielsenSet2. As TEPITOPEpan did not need any training data, we ran TEPITOPEpan directly on NielsenSet2 to get its prediction result [15]. For all other models, the experimental result was achieved by LOO, where we trained the model on the binding peptides of 13 alleles, and then made prediction on the one allele left as testing [10,11]. The results of MultiRTA, NetMCHIIpan-2.0 and NetMHCIIpan-1.0 were from [11,14]. For MHC2SKpan, we learned the model using the parameters that achieved the best average AUC per allele in the training data, and made prediction on the test allele. The experimental results show that NetMHCIIpan-2.0 and MHC2SKpan are two best prediction methods with very close performances. For example, NetMHCIIpan-2.0 achieved the highest average PCC of 0.606, which is closely followed by MHC2SKpan (0.605), and then NetMHCIIpan-1.0 (0.541), MultiRTA (0.531), and TEPITOPEpan (0.404). Specifically, MHC2SKpan outperformed NetMHCIIpan-2.0 in 8, NetMHCIIpan-1.0 in 13, MultiRTA in 12, and TEPITOPEpan in 14 out of all 14 alleles, with last three being statistically significant (binomial test, *p*-value < 0.05). Similar experimental results were obtained in terms of AUC. NetMHCIIpan-2.0 obtained the largest average AUC of 0.799, which is closely followed by MHC2SKpan (0.795), and then MultiRTA (0.773), NetMHCIIpan-1.0 (0.767), and TEPITOPEpan (0.710). Specifically, MHC2SKpan outperformed NetMHCIIpan-2.0 in 6, MultiRTA in 11, NetMHCIIpan-1.0 in 12, and TEPITOPEpan in 13 out of all 14 alleles. The last three are statistically significant (binomial test, *p*-value < 0.05). Overall, MHC2SKpan outperformed NetMHCIIpan-1.0, MultiRTA and TEPITOPEpan, being statistically significant, and achieved the comparable performance with the state-of-the-art predictor, NetMHCIIpan-2.0.

**Table 2:** LOO benchmark comparison of MHC2SKpan with four well-known pan-specific methods on NielsenSet2. MRTA, Tepan, Pan1.0, Pan2.0 and MKpan are the abbreviations for MultiRTA, TEPITOPEpan, MetaMHCIIpan-1.0, MetaMHCIIpan-2.0 and MHC2SKpan, respectively. For each allele, we display the largest value in boldface.

		AUC					PCC				
**allele**	**count**	**MRTA**	**Tepan**	**Pan1.0**	**Pan2.0**	**MKpan**	**MRTA**	**Tepan**	**Pan1.0**	**Pan2.0**	**MKpan**

DRB1*01:01	5166	0.801	0.726	0.778	0.794	**0.802**	0.619	0.447	0.571	0.627	**0.628**
DRB1*03:01	1020	0.751	0.663	0.746	**0.792**	0.778	0.438	0.277	0.465	**0.560**	0.543
DRB1*04:01	1024	0.763	0.724	0.775	**0.802**	0.801	0.534	0.423	0.591	0.652	**0.657**
DRB1*04:04	663	0.835	0.783	0.852	**0.869**	0.862	0.623	0.504	0.693	**0.731**	0.714
DRB1*04:05	630	0.808	0.760	0.808	0.823	**0.828**	0.566	0.456	0.594	0.626	**0.631**
DRB1*07:01	853	0.817	0.759	0.825	0.886	**0.889**	0.620	0.499	0.655	0.753	**0.761**
DRB1*08:02	420	0.786	0.773	0.841	**0.869**	0.851	0.523	0.452	0.637	**0.70**	0.679
DRB1*09:01	530	0.674	0.615	0.653	**0.684**	0.674	0.380	0.259	0.406	**0.474**	0.471
DRB1*11:01	950	0.819	0.726	0.799	0.875	**0.894**	0.603	0.450	0.580	0.721	**0.761**
DRB1*13:02	498	**0.698**	0.661	0.658	0.648	0.639	**0.365**	0.326	0.323	0.337	0.341
DRB1*15:01	934	0.729	0.694	0.738	**0.769**	0.763	0.513	0.437	0.533	**0.598**	0.597
DRB3*01:01	549	**0.813**	0.675	0.716	0.733	0.70	**0.603**	0.332	0.449	0.474	0.423
DRB4*01:01	446	0.746	0.694	0.724	0.762	**0.764**	0.508	0.370	0.448	0.515	**0.529**
DRB5*01:01	924	0.788	0.680	0.831	0.879	**0.883**	0.543	0.421	0.627	0.722	**0.737**

average	14607	0.773	0.710	0.767	**0.799**	0.795	0.531	0.404	0.541	**0.606**	0.605

### Evaluation by NielsenSet3

Table 3 compares the performance of MHC2SKpan with TEPITOPEpan and NetMHCIIpan-2.0 on NielsenSet3 using 5-fold cross validation. The partition of the data, and the experimental result of NetMHCIIpan-2.0 are from the original paper [11]. As NetMHCIIpan-1.0 and MultiRTA were not trained on NielsenSet3 using 5-fold cross-validation, we could not report their results in Table 3. We ran TEPITOPEpan directly on NielsenSet3 to get its prediction result [15]. From this experimental result using 5-fold cross validation, we can find again that MHC2SKpan achieved comparable performance with NetMHCIIpan-2.0. Since TEPITOPEpan could not take advantage of sufficient training data, it did not perform very well. For example, NetMHCIIPan-2.0 achieved an average AUC of 0.846, and MHC2SKpan achieved an AUC of 0.843, which was followed by TEPITOPEpan (0.738). Specifically, MHC2SKpan outperformed NetMHCIIpan-2.0 in 11, and TEPITOPEpan in 23 out of 24 alleles. And the last one is statistically significant (binomial test, *p*-value < 0.01).

**Table 3:** Five-fold cross validation comparison of MHC2SKpan and NetMHCIIpan-2.0 on NielsenSet3. For each allele, we display the largest value in boldface.

		AUC			PCC		
**allele**	**count**	**TEPITOPEpan**	**NetMHCIIpan-2.0**	**MHC2SKpan**	**TEPITOPEpan**	**NetMHCIIpan-2.0**	**MHC2SKpan**

DRB1*01:01	7685	0.731	**0.846**	0.845	0.433	**0.711**	0.702
DRB1*03:01	2505	0.718	**0.864**	0.853	0.346	**0.709**	0.672
DRB1*03:02	148	0.603	**0.757**	0.755	0.227	**0.525**	0.447
DRB1*04:01	3116	0.765	**0.848**	0.840	0.438	**0.670**	0.647
DRB1*04:04	577	0.758	**0.818**	0.816	0.496	**0.630**	0.622
DRB1*04:05	1582	0.783	0.858	**0.869**	0.491	0.698	**0.703**
DRB1*07:01	1745	0.781	0.864	**0.872**	0.533	0.740	**0.742**
DRB1*08:02	1520	0.650	0.780	**0.784**	0.294	0.526	**0.532**
DRB1*08:06	118	0.870	**0.924**	0.912	0.602	**0.796**	0.749
DRB1*08:13	1370	0.747	0.885	**0.896**	0.337	0.746	**0.760**
DRB1*08:19	116	0.714	0.808	**0.831**	0.537	0.608	**0.623**
DRB1*09:01	1520	0.683	0.818	**0.826**	0.340	0.634	**0.638**
DRB1*11:01	1794	0.797	**0.883**	0.877	0.514	**0.777**	0.764
DRB1*12:01	117	0.831	**0.892**	0.876	0.627	**0.764**	0.754
DRB1*12:02	117	0.843	**0.900**	0.898	0.640	**0.769**	0.762
DRB1*13:02	1580	0.602	**0.825**	0.811	0.238	**0.634**	0.591
DRB1*14:02	118	0.724	0.860	**0.889**	0.445	0.694	**0.735**
DRB1*14:04	30	0.683	**0.737**	0.621	0.489	**0.613**	0.418
DRB1*14:12	116	0.805	0.894	**0.904**	0.517	**0.757**	0.742
DRB1*15:01	1769	0.739	0.819	**0.834**	0.465	0.653	**0.669**
DRB3*01:01	1501	0.671	**0.850**	0.832	0.289	**0.690**	0.636
DRB3*03:01	160	0.771	0.853	**0.864**	0.403	**0.736**	0.702
DRB4*01:01	1521	0.685	0.837	**0.861**	0.351	0.675	**0.712**
DRB5*01:01	3106	0.764	**0.882**	0.875	0.445	**0.765**	0.736

average	33931	0.738	**0.846**	0.843	0.437	**0.688**	0.669

### Evaluation by EpanSet4

Table 4 compares the performance of MHC2SKpan and other four pan-specific methods on an independent testing set, EpanSet4. Please note that 12 out of all 14 alleles are not in any of NielsenSet1, NielsenSet2 and NielsenSet3, which means that it is a good benchmark dataset for examining the performance of pan-specific models on novel alleles. MHC2SKpan was trained on NielsenSet3 using LOO, and the result of other pan-specific methods are from the original paper [15]. From the experimental results we find that MHC2SKpan performed best among all five pan-specific methods. MHC2SKpan obtained the largest average AUC (0.734), which is followed by NetMHCIIpan-2.0 (0.732), TEPITOPEpan (0.712), NetMHCIIpan-1.0 (0.701) and MultiRTA (0.677). MHC2SKpan outperformed both NetMHCIIpan-2.0 and NetMHCIIpan-1.0 in 9, and MultiRTA in 11 out of all 14 alleles. If we exclude two molecules (DRB1*12:01 and DRB1*03:02) appearing in NielsenSet3, we can still see clear advantage of MHC2SKpan over other pan-specific methods. In this case, MHC2SKpan obtained the largest average AUC of 0.730, which is followed by NetMHCIIpan-2.0 (0.722), TEPITOPEpan (0.707), NetMHCIIpan-1.0 (0.693) and MultiRTA (0.672).

**Table 4:** The AUC performance comparison of MHC2SKpan with MutliRTA, TEPITOPEpan, NetMHCIIpan-1.0 and NetMHCIIpan-2.0 on EpanSet4. For each allele, we display the largest value in boldface. The last row is the average result by excluding two alleles in NielsenSet3, DRB1*03:02 and DRB1*12:01.

allele	count	MultiRTA	TEPITOPEpan	NetMHCIIpan-1.0	NetMHCIIpan-2.0	MHC2SKpan
DRB1*01:02	92	0.749	0.758	**0.785**	0.746	0.752
DRB1*01:03	52	0.772	**0.867**	0.756	0.772	0.798
DRB1*03:02	88	0.733	0.823	0.775	**0.840**	0.761
DRB1*04:03	63	0.611	**0.762**	0.659	0.678	0.714
DRB1*04:06	92	0.519	0.501	**0.557**	0.486	0.489
DRB1*11:02	65	0.591	0.738	0.738	**0.774**	0.766
DRB1*11:03	64	0.585	0.726	0.623	**0.791**	0.785
DRB1*11:04	73	0.618	0.654	0.639	0.737	**0.740**
DRB1*12:01	719	0.673	0.659	0.721	0.740	**0.753**
DRB1*13:01	302	0.567	**0.623**	0.516	0.494	0.485
DRB1*14:01	43	**0.809**	0.785	0.761	0.676	0.721
DRB1*15:02	47	0.777	0.742	0.762	0.888	**0.899**
DRB1*16:01	56	0.789	0.644	0.793	0.814	**0.817**
DRB3*02:02	656	0.680	0.686	0.732	**0.806**	0.789

Average	2412	0.677	0.712	0.701	0.732	**0.734**
Average*	1605	0.672	0.707	0.693	0.722	**0.730**

**Figure 1:**
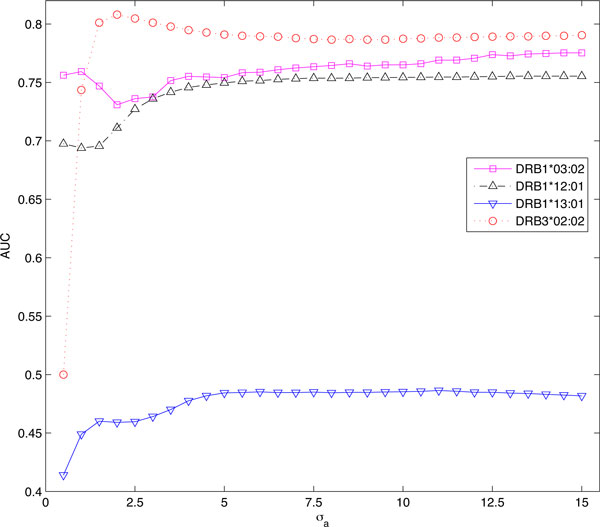
**The performance of MHC2SKpan under different setting of σ*a***. The performance of MHC2SKpan on DRB1*03:02, DRB1*12:01, DRB1*13:01 and DRB3*02:02 in EpanSet4 under different settings of *σ_a_*.

In this experiment, MHC2SKpan used the same set of parameters to predict the binding specificities of novel alleles. The parameters were estimated from training data NielsenSet3 using LOO, and it might not be a good configuration for a novel allele. The parameter σ*a *of MHC2SKpan is actually used to measure the similarities among different MHC molecules. A large *σ_a _*will incorporate the binding data of more MHC molecules into training process, and it may bring some unrelated MHC molecules. On the other hand, a small *σ_a _*will only incorporate the binding data of a small number of MHC molecules into training process, and it may omit some related MHC molecules. In an ideal case, a suitable *σ_a _*should be used for each target MHC molecule. To examine the effect of *σ_a_*, we further checked the performance of MHC2SKpan on the 4 DRB alleles in EpanSet4: DRB1*12:01, DRB3*02:02, DRB1*13:01 and DRB1*03:02. The reason for choosing these four alleles was that (1) they have large number of binding data (DRB1*12:01, DRB3*02:02 and DRB1*13:01); or (2) they do not appear in NielsenSet3 (DRB1*03:02 and DRB1*12:01). Figure 1 shows the change of AUC on these 4 alleles with respect to the variation of *σ_a_*. Here *σ_a _*ranges from 0.5 to 15 with an interval of 0.5. *σ_a _*= 6.5 is the learned parameter from NielsenSet3 used to generate Table 4. We can see that it is actually not a good setting for these alleles, especially for DRB3*02:02. Specifically, for DRB3*02:02, the best AUC is 0.808 with *σ_a _*= 2 which is much higher than its current performance (0.789) under default setting. Another interesting discovery is that, for DRB1*03:02, with a large σ*a*, the performance is actually improved. This may suggest more binding data from other alleles is helpful for DRB1*03:02. All these indicate that the performance of MHC2SKpan could be further improved if we can customize the parameters for the target MHC molecules.

## Discussion

Both GS and MHC2SK have their roots in SRBF, which only considers substrings of a fixed length for computing similarities. However, by considering the characteristics of MHC-II peptide binding prediction, MHC2SK explicitly incorporates two important features into the kernel design: (1) emphasizing more on long substrings and (2) the great variation of peptide lengths. In contrast, without considering these domain knowledge, GS has to tune an additional parameter *σ_p_*, which will increase training cost heavily. It may also lead to unsatisfactory result due to scarcity and noisy in training data. The experimental results on NielsenSet1 clearly demonstrate the advantage of MHC2SK over GS and SRBF. Actually, incorporating domain knowledge into model design becomes increasingly important for achieving the good prediction accuracy in bioinformatics [32].

Furthermore, we extend MHC2SK to MHC2SKpan for pan-specific MHC binding prediction. The performance of MHC2Skpan and other four well known pan-specific methods have been extensively evaluated using three benchmark datasets by LOO, cross-validation and independent testing. MHC2SKpan achieved good performance in all these experiments. Specifically, the LOO result on NielsenSet2 shows that MHC2SKpan outperformed NetMHCIIpan-1.0, TEPITOPEpan and MultiRTA, being statistically significant. MHC2SKpan achieved comparable performance with the-state-of-the-art model, NetMHCIIpan-2.0, in both LOO on NielsenSet2 and 5-fold cross validation on NielsenSet3. Moreover, MHC2SKpan is the best method in the independent test on EpanSet4. Experimental results also suggest that MHC2SKpan can achieve better prediction result if we customize the parameters for the target MHC molecules. Additionally, in contrast to NetMHCIIpan-2.0 using ensemble techniques, MHC2SKpan is an individual model. The performance of MHC2SKan could be further improved by various ensemble techniques [33,34].

## Conclusion

In this work, we present a state-of-the-art kernel based method, MHC2SKpan, for pan-specific MHC-II binding prediction. On the one hand, it can effectively incorporate the physical and chemical properties of amino acids for measuring the similarities among the peptides of different lengths. On the other hand, the relationship among different MHC molecules can be directly captured and utilized for pan-specific binding prediction. Experimental results on various benchmark datasets from different perspectives demonstrated that MHC2SKpan achieved comparable performance with the leading predictor, NetMHCIIpan-2.0, and outperformed three well known pan-specific methods, NetMHCIIpan-1.0, TEPITOPEpan and MultiRTA, being statistically significant. Automatically tuning the parameters in MHC2SKpan for a novel target MHC to improve its performance would be a very interesting future work.

## Competing interests

The authors declare that they have no competing interests.

## Authors' contributions

Method development: LG SZ. Conceived and designed the experiment: LG SZ. Performed the experiment: LG CL. Designed the web site LG. Analyzed the data: LG SZ. Wrote the paper: LG SZ.

## Declarations

Publication of this article was funded by National Natural Science Foundation of China.

This article has been published as part of *BMC Genomics *Volume 14 Supplement 5, 2013: Twelfth International Conference on Bioinformatics (InCoB2013): Computational biology. The full contents of the supplement are available online at http://www.biomedcentral.com/bmcgenomics/supplements/14/S5.
